# Tracking financing for global common goods for health: A machine learning approach using natural language processing techniques

**DOI:** 10.3389/fpubh.2022.1031147

**Published:** 2022-11-17

**Authors:** Siddharth Dixit, Wenhui Mao, Kaci Kennedy McDade, Marco Schäferhoff, Osondu Ogbuoji, Gavin Yamey

**Affiliations:** ^1^Center for Policy Impact in Global Health, Duke Global Health Institute, Duke University, Durham, NC, United States; ^2^Open Consultants, Berlin, Germany

**Keywords:** classification, natural language processing, machine learning, global common goods for health, official development assistance

## Abstract

**Objective:**

Tracking global health funding is a crucial but time consuming and labor-intensive process. This study aimed to develop a framework to automate the tracking of global health spending using natural language processing (NLP) and machine learning (ML) algorithms. We used the global common goods for health (CGH) categories developed by Schäferhoff et al. to design and evaluate ML models.

**Methods:**

We used data curated by Schäferhoff et al., which tracked the official development assistance (ODA) disbursements to global CGH for 2013, 2015, and 2017, for training and validating the ML models. To process raw text, we implemented different NLP techniques, such as removing stop words, lemmatization, and creation of synthetic text, to balance the dataset. We used four supervised learning ML algorithms—random forest (RF), XGBOOST, support vector machine (SVM), and multinomial naïve Bayes (MNB) (see Glossary)—to train and test the pre-coded dataset, and applied the best model on dataset that hasn't been manually coded to predict the financing for CGH in 2019.

**Results:**

After we trained the machine on the training dataset (*n* = 10,534), the weighted average F1-scores (a measure of a ML model's performance) on the testing dataset (*n* = 2,634) ranked 0.79–0.83 among four models, and the RF model had the best performance (F1-score = 0.83). The predicted total donor support for CGH projects by the RF model was $2.24 billion across 3 years, which was very close to the finding of $2.25 billion derived from coding and classification by humans. By applying the trained RF model on the 2019 dataset, we predicted that the total funding for global CGH was about $2.7 billion for 730 CGH projects.

**Conclusion:**

We have demonstrated that NLP and ML can be a feasible and efficient way to classify health projects into different global CGH categories, and thus track health funding for CGH routinely using data from publicly available databases.

## Key messages

### What is already known on this topic

- Estimating global health financing flows is an essential foundation for advocacy efforts and policymaking.
- There have been many efforts to improve the transparency of the flows of official development assistance (ODA), including establishing publicly available databases of ODA, such as the Organization for Economic Cooperation and Development (OECD) Creditor Reporting System (CRS).
- However, such databases do not capture all the targets of global health spending, such as global common goods for health (CGH). Tracking CGH is a time consuming and labor-intensive process that involves reading detailed project reports and manually categorizing individual aid activities.

### What this study adds

- We developed a machine learning architecture to track the global financing for CGH and compared the machine classified CGH projects with human classifications. We then applied the trained machine learning algorithm to predict the global financing for CGH.
- After we trained the machine on the training dataset (*n* = 10,534), the weighted average F1-scores on the testing dataset (*n* = 2,634) ranked 0.79–0.83 among four models. The predicted total donor support for CGH projects by the RF model was $2.24 billion across 3 years, which was very close to the finding of $2.25 billion derived from coding and classification by humans.
- By applying the trained RF model on the 2019 dataset, we predicted the total funding for global CGH was about $2.7 billion for 730 CGH projects.

### How this study might affect research, practice or policy

- We have demonstrated that NLP and ML can be a feasible and efficient way to classify health projects into different global CGH categories, and thus track health funding for CGH routinely using data from publicly available databases.
- By tracking the global financing for CGH, we provided evidence to support the advocacy efforts on increasing the funding for CGH.

## Introduction

Estimating global health financing flows is an essential foundation for advocacy efforts and policymaking. Reliable estimates enable evidence-based decision making and identify critical financing gaps in the global health architecture. There have been many efforts to improve the transparency of the flows of official development assistance (ODA), including establishing publicly available databases of ODA, such as the Organization for Economic Cooperation and Development (OECD) Creditor Reporting System (CRS).

However, such databases do not capture all the targets of global health spending, and therefore researchers must come up with innovative ways to estimate the amount of global health funding that targets specific purposes. One example is funding for global common goods for health (CGH)—defined as activities that provide transnational health benefits, such as pandemic preparedness and response, knowledge generation and sharing, and tackling antimicrobial resistance. The WHO published a special series in 2019 highlighting the importance of funding common goods (1). One paper in the series by Schäferhoff et al. attempted to estimate ODA disbursements to global CGH in three different years (2013, 2015, and 2017) (2). The estimates, while robust, required a very labor-intensive process of reading detailed project reports and manually categorizing individual aid activities. Machine learning methods could be a powerful tool to help such investigations, providing extra automated quality control and eventually allowing estimations over a longer time period with minimal additional labor inputs.

One example of applying machine learning in estimating and categorizing financial flows is by the Institute for Health Metrics and Evaluation (IHME). In 2020, IHME published a report on the impact of COVID-19 on financing global health (3). The report used machine learning (ML) and natural language processing (NLP) (see Box 1, Glossary) to make predictions for how the pandemic would affect development assistance for health. Moreover, many studies in healthcare have used ML and NLP to automate different tasks or find patterns in large datasets. For example, Weikert et al. used NLP techniques as well as convolutional neural network (CNN), support vector machine (SVM), and random forest (RF) algorithms to classify unstructured radiology reports, obtaining a high accuracy (16). Kim et al. applied NLP techniques based on deep learning (neural networks) to find information from online resources and articles to quickly identify potential outbreaks of infectious disease (17). The authors were able to achieve an accuracy of above 90% for all the ML models in identifying infectious disease outbreaks.

Box 1 **Glossary of terms.**

**Machine learning:** Machine Learning is the science (and art) of programming computers so they can learn from data (4).

**Natural language processing:** Natural language processing is the study of computer programs that take natural, or human, language as the input (5).

**Supervised learning:** Supervised learning is a subcategory of machine learning. It is defined by its use of labeled datasets to train algorithms to classify data or predict outcomes accurately (6).

**Support vector machines:** A set of supervised learning methods used for classification, regression and detection of outliers (7).

**Random forest classifiers:** Random forest is a commonly-used supervised machine learning algorithm that combines the output of multiple decision trees to reach a single result (8).

**Multinomial naïve Bayes (MNB):** A Bayesian classifier and is often used for text classification (9).

**XgBoost:** Stands for extreme gradient boosting and is an optimized distributed gradient boosting machine learning algorithm under gradient boosting framework (10).

**Deep learning:** A specific subfield of machine learning: a new take on learning representations from data that puts an emphasis on learning successive layers of increasingly meaningful representations (11).

**Neural networks:** In deep learning, the layered representations are learned *via* models called neural networks, structured in literal layers stacked on top of each other (11).

**Convolutional neural network:** A class of deep learning methods that has become dominant in various computer vision tasks (12).

**Feature engineering:** The process of using the domain knowledge to come up with good sets of features for machine learning algorithm to train (4).

**Vectorization:** A process that enables machines to understand raw data by converting them into meaningful numerical representations (13).

**Tokenization:** The process of breaking down raw text into tokens i.e., words, characters, etc.

**Lemmatization:** The use of a vocabulary and morphological analysis of words, normally aiming to remove inflectional endings only and to return the base or dictionary form of a word, which is known as the lemma (14).

**Cross-validation:** A statistical method of evaluating generalization performance that is more stable and thorough than using a split into a training set and a test set. In cross validation, the data are split repeatedly, and multiple models are trained. The most commonly used version of cross-validation is k-fold cross-validation, where k is a user-specified number, usually 5 or 10 (4).

**Precision:** Attempts to answer the question “what proportion of positive identifications was actually correct?” (15)

**Recall:** Attempts to answer the question, “what proportion of actual positives was identified correctly?” (15)

**F1 score:** The harmonic mean of precision and recall (4).

However, getting quality training data is always a challenge in ML projects. The dataset created by Schäferhoff et al. provided us with a readily available resource to test our hypothesis– whether NLP and ML tools could be used to help track CGH funding. In this study, we aimed to design and implement a ML pipeline based on NLP techniques to automate Schäferhoff et al.s' manual categorization method for estimating funding for CGH (2) (Box 2). We then applied the newly developed ML framework to the 2019 OECD CRS database to estimate funding (ODA disbursements) for CGH in that year.

Box 2 **Schäferhoff et al.s' manual categorization method for estimating funding for CGH.**

Schäferhoff et al. tracked international funding for CGH using the taxonomy developed by The Lancet Commission on Investing in Health, which divided global functions into three broad categories: provision of global public goods for health, managing negative regional and global externalities, and fostering leadership and stewardship (18). Schäferhoff et al. further divided these three categories into 11 categories of CGH (Annex 1). They used the OECD CRS database to access information about projects funded by health aid. The authors downloaded projects with purpose codes for “aid to health”: “health, general (purpose code 121),” “basic health (purpose code 122),” and “population policies/programmes and reproductive health (purpose code 130)”. They also accessed humanitarian aid purpose codes (72010, 72040, 72050, 73010, and 74010) to make sure that any funding for epidemic and pandemic preparedness and response (a critical CGH) was also included in the analysis. To understand time trends in funding for CGH, Schäferhoff et al. downloaded projects for the years 2013, 2015, and 2017 from the OECD CRS database that had the above-mentioned purpose codes.

The authors manually classified the projects from these 3 years into 11 CGH categories. They then calculated the amount of funding that was targeted at each of these 11 categories.

## Methods

**Figure 1:**
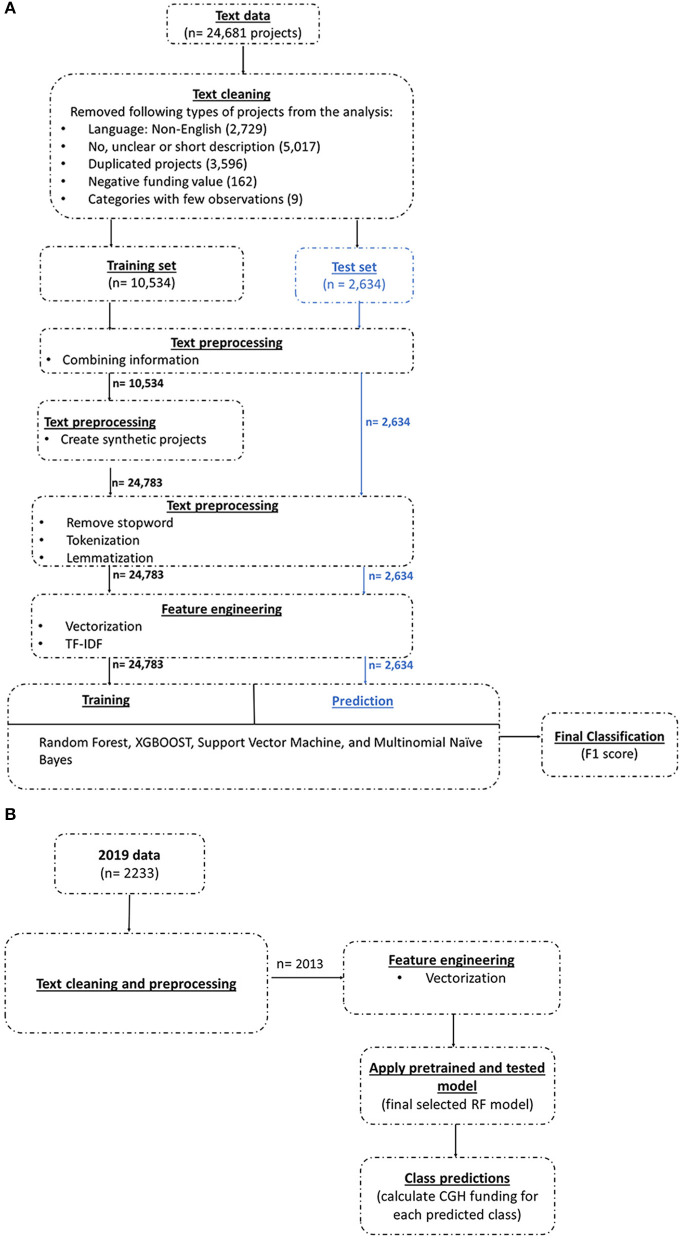
**(A)** Machine learning pipeline for global CGH classification. **(B)** Machine learning pipeline for predicting CGH categories in 2019 data.

The three main steps of a ML approach are training, validating and testing the ML model. Figure 1A captures the pipeline of the machine learning approach implemented in the current analysis. We describe each step in more detail below.

Our first step was to use a dataset that was classified manually to train and validate the ML models. We used data from 2013, 2015, and 2017 from the OECD CRS database obtained from Schäferhoff et al. to train the model. We applied the data preprocessing and cleaning steps shown in Figure 1A. Then we explored four ML models to train on the pre-processed data. Once the chosen models were trained and validated, we measured the performance of the validated models on a set of test data, previously unseen by these ML algorithms, to evaluate and understand their performance when applied to real world data. Finally, we applied the trained model with the highest weighted average F1 score to a new 2019 dataset obtained from the OECD CRS database, unseen by the trained model, to estimate the funding for CGH in that year (see Box 1, Glossary).

### Data collection

We collected two types of data from the OECD CRS database:

#### Pre-coded data

For training, validating, and testing the model, we used the dataset created by Schäferhoff et al. that was originally extracted from the OECD CRS database (19, 20), using the health focused purpose codes. The initial dataset that we obtained from Schäferhoff et al. included 24,681 projects with information on financing by funders for health-related projects in 2013, 2015, and 2017. For this new study, we used an earlier version of Schäferhoff et al.'s dataset—the number of projects in this earlier version is not exactly the same as in the final version. The small discrepancy is unlikely to affect our results.

#### Data for application

We extracted 2019 health-related data following the strategy outlined by Schäferhoff et al. (Annex 1; Box 2). This step was an application of the final selected model (the best performing model on the test data) on a new dataset—one that had not been manually coded. The downloaded data included information on the donor's name, recipient name, purpose code, project long and short description, project title, and funding amount for each project.

As noted in Annex 1, we included projects under the purpose code 16064 (“social mitigation of HIV”), and under the humanitarian purpose codes 72010, 72040, 72050, 73010, and 74020. Projects with these five humanitarian purpose codes were included in order to capture all funding for epidemic and pandemic response (not just funding *via* the health sector).

#### Definitions

We used Schäferhoff et al.'s eleven categories of CGH (2) for training and testing the ML models. In addition to the eleven categories, we created an extra category called “not a global function”. This category included projects that did not fall into any of the predefined eleven classes. The eleven categories of global CGH were (1) R&D of new health tools; (2) development and harmonization of international health regulations; (3) knowledge generation and sharing; (4) sharing of intellectual property; (5) market shaping activities; (6) epidemic and pandemic preparedness and response; (7) responses to antimicrobial resistance; (8) responses to marketing of unhealthful products; (9) control of cross border disease movement; (10) health advocacy and priority setting; and (11) promotion of aid effectiveness and accountability (further details can be found in Annex 2).

### Data/text cleaning, splitting, and preprocessing

We applied several data cleaning and preprocessing steps to prepare the data for the analysis. These steps were applied to both the pre-coded data used for training and testing the models, and the 2019 data used for application of the pretrained model.

#### Data cleaning

We removed five types of projects (*n* = 11,513) from the analysis (Figure 1A):

*(1) Language:* For the current analysis, we focused only on projects described in the English language. Therefore, we removed all the projects that had a project description in a language other than English (*n* = 2,729).
*(2) No, unclear, or short description:* We excluded projects with no, an unclear or only a short text description for a manual coder to classify the projects (*n* = 5,017).
*(3) Duplicated projects:* We dropped duplicated projects as these would not provide new knowledge to the ML model (*n* = 3,596).
*(4) Negative funding value:* In the dataset, some projects had a negative funding amount as the loan repayments for these projects are higher than new ODA. We excluded these projects from the analysis (*n* = 162) (21).
*(5) Categories with few observation/projects:* An imbalance in the number of projects among different categories could result in biased predictions toward a category with a large number of projects (22). For example, “Knowledge generation and sharing” had the largest number of observations with 4,003 projects, whereas “Epidemic and pandemic preparedness and response” and “R&D of new health tools”, were the second and third largest CGH categories with 1,795, and 1,706 projects, respectively (Annex 3). We decided to drop “Sharing of intellectual property” (*n* = 9) and “Responses to antimicrobial resistance (*n* = 0)” from the database as the ML algorithm would not be able to learn anything meaningful from these classes.

#### Splitting datasets into separate training and testing sets

After data cleaning, 13,168 projects were eligible for analysis and were randomly divided into two datasets: 80% (*n* = 10,534) went into the training set and 20% (*n* = 2,634) into the test set.

A training dataset was used to train the ML model and inform its future predictions. We included a large portion of the projects in the training set so that there would be a diverse dataset to train the ML models, which could increase the prediction accuracy.

The text pre-processing steps were implemented separately on the training and testing datasets (Figure 1A). The steps were implemented separately to avoid information leakage to the ML model—to make sure that the ML algorithm only saw the training dataset, and the test dataset remained completely unknown to the algorithm. Finally, the test set was used to gauge the performance of the chosen model.

#### Pre-processing

We included pre-processing steps, including combining information from different columns in the dataset and creating synthetic text to balance the data in each CGH category.

We also undertook several standard procedures in NLP tasks, including feature engineering, tokenization, punctuations and stop word removal, lemmatization and vectorization (see Box 1, Glossary) (23, 24). These standard steps should be implemented before executing a model for it to learn meaningful information from the data. Figure 1A shows the preprocessing steps undertaken on the training and test datasets, respectively. More information on each data preprocessing step is given below:

*(1) Combining information:* Project title, purpose code, long and short descriptions carry relevant information from which a ML model can learn about the project. Therefore, we concatenated all these variables to create a single description and used the combined text for training the model. There was no change in the number of projects in each subcategory after the implementation of this preprocessing step.
*(2) Creating synthetic projects/text:* After the data cleaning steps, an imbalance in the number of projects among different categories remained (see Annex 4). We applied the synonym replacement technique to create synthetic text to balance the categories and thus reduce the potential bias caused by imbalanced data (25). Specifically, we created artificial projects by replacing certain words with their synonyms in the original project description (26, 27). This step created new observations using the already available information in the original project descriptions. The synthetic text was created only for the training dataset. For the training set we created 14,249 new projects using this technique to balance the dataset. As a result, the total number of projects in the training data increased to 24,783.
*(3) Feature engineering:* In addition to the already existing features in the training data, new features can be created and added to the dataset to help the ML classifiers to learn the underlying structure of text description in each category. New features can be created based on expert knowledge about the data which could help in differentiating projects into different categories. Based on our understanding of the dataset, we created three new features for each project description–number of characters, number of sentences, and the percentage of punctuations.
*(4) Tokenization:* By this stage, we had project descriptions as text, which was a collection of words, punctuations, and white spaces. But for the ML algorithm, these are just a collection of one long string of characters, which does not provide any useful information. Therefore, we tokenized the project description i.e., we split the text in each project description by the white spaces. Now, instead of having a long string of text, we had a list of tokens (without any white spaces), which helps the model to learn better from each word.
*(5) Removing punctuations and stop words*: After we tokenized the text, we got a list of tokens for each project description. However, some words or tokens could be more important than the other. For example, words such as *the, am, a, an, and, of*, *or*, etc. appeared very frequently in any text but did not add much to the information. These words are stop words and we removed stop words so as to let the ML algorithm focus more on pivotal words in the project description. Removing the stop words still captured the important information in the sentence but reduced the number of tokens that the algorithm had to look at to capture the relevant information. The same argument applied for the removal of punctuation from the text as its deletion did not impact the information available in the remaining tokens.
*(6) Lemmatization*: After removing punctuations and stop words, we implemented lemmatization (14). The process of lemmatization helped the algorithm understand that words such as *grew, grow* and *growing* all have the same semantic meaning. Therefore, with lemmatization we replaced the words with same semantic meaning into their base word. This helped in reducing the corpus of words that the ML model was exposed to, in order to explicitly correlate words with similar meaning. This reduced the number of tokens that an algorithm needs to learn from and allowed it to focus on the most pivotal words in the text.
*(7) Vectorization of the raw text:* We had a list of tokens that the model could use to learn from. But the algorithm still only took them as characters, and it was not able to learn from a list of characters. Therefore, we converted the text tokens into a format that an algorithm could use to build a model. This process is called vectorizing. Vectorizing encodes the text as integers to create feature vectors.

A feature vector is a n-dimensional vector of numerical features that represents some object. Simply stated, we took text tokens of each project description and converted them into a numeric vector that represents this text in a way the algorithm can understand and use to train the model. In our analysis, we used the Term frequency-inverse document frequency (TF-IDF) method for vectorizing the text for each project as this is the most widely used algorithm (28, 29). TF-IDF creates a document term matrix, where there is one row for each project description, and the columns represent single unique terms/tokens. Each cell in this matrix has a number that represents a weighting that identifies how important a word is to each project description. The following formula shows how the weighting of each word in the text is determined:


$$\begin{array}{l} w_{i,j}=tf_{i,j}*\log\left(\frac{N}{df_{i}}\right) \end{array}$$

*tf*_*i, j*_= number of times i occurs in j divided by total number of terms in j

*df*_*i*_ = number of documents containing i

*N* = total number of documents

We used R (The R Foundation, Vienna, Austria), RStudio (RStudio, Boston, Massachusetts), and Python for data pre-processing and analysis.

### Data analysis/classification

#### Training

For training, we explored four supervised learning algorithms—random forest (RF), XGBOOST, support vector machine (SVM), and multinomial naïve bayes (MNB) (10, 30). We also implemented 5-fold cross validation for performance evaluation during the model training, and for parameter tuning of various ML algorithms. Accuracy, precision, recall, and F1-score are generally used statistical tests for validating models in a multiclass classification task (31). To balance both precision and recall for our final prediction, we used the weighted average F1-score as the metric to compare and pick the best ML model for the current multiclass classification task.

#### Testing

After training and validation, we applied the selected ML model on the 20% unseen projects in the test dataset as the final step in model evaluation. Except for the data preprocessing step of creating synthetic text, all other steps were implemented separately on both the training set and test set (Figure 1A). Synthetic text was only created to balance the training dataset, and we kept the test data as completely unseen. Once the test data were classified using the selected model, we calculated the aggregated amount of funding for each CGH category and compared the ML classified results with those coded manually.

### Application of pretrained, and tested model

The last step of this study was to apply the pretrained and tested ML model to the 2019 dataset that was not coded manually. The purpose of this final step was to estimate the funding for CGH using the pretrained and tested ML model. We downloaded the health projects for 2019 for the same purpose codes as described in the methods sections. We excluded non-communicable disease (NCD) projects from the 2019 data as these projects were not available in the training dataset. However, it is technically feasible to include the latest purpose code in the training dataset to retrain the model in future analyses.

There were 2,233 projects in the 2019 dataset. We followed most of the same data processing procedure as undertaken for the test dataset. After the preprocessing step we included 2,013 projects in the analysis. We used the selected pretrained and tested model to classify the projects in the 2019 data into different CGH categories. Figure 1B shows the pipeline of predicting CGH classifications for the unseen 2019 dataset using a pretrained and tested ML model.

## Results

The cleaned dataset with 13,168 projects comprised of 10 categories—nine CGH and one “not a global function” category. In the first part of the results section below, we report findings from the training and testing of ML models, including the F1-scores for the four ML algorithms and comparison between the human and machine classified CGH funding. In second part, we report prediction of CGH classifications for 2019 using the pretrained and tested ML model.

### Part 1: Training and testing of ML models

We trained the four ML models on the training dataset and tested the performance on the test datasets. Table 1 shows the F1-scores for the four ML algorithms for each CGH category on the test dataset. The total average weighted F1-score on the test dataset was highest for the RF and XgBOOST algorithms (0.83), indicating better performance. RF was finally selected for the final application to 2019 data because it takes less time to train.

**Table 1:** F1-scores^a^ of RF, XgBOOST, SVM, and MNB.

**Categories (number of projects)**	**RF**	**XgBOOST**	**SVM**	**MNB**
R&D of new health tools (330)	0.73	0.74	0.73	0.72
Development/ harmonization of international health regulations (53)	0.82	0.85	0.79	0.76
Knowledge generation and sharing (585)	0.76	0.78	0.76	0.73
Market shaping activities (224)	0.96	0.97	0.96	0.93
Epidemic and pandemic preparedness and response (328)	0.88	0.89	0.86	0.82
Responses to marketing of unhealthful products (60)	0.81	0.86	0.80	0.77
Control of cross border disease movement (310)	0.95	0.94	0.91	0.85
Health advocacy and priority setting (203)	0.91	0.90	0.88	0.87
Promotion of aid effectiveness and accountability (57)	0.48	0.48	0.56	0.65
Not a global function (484)	0.80	0.80	0.78	0.77
Total weighted average (2,634)	**0.83**	**0.83**	**0.81**	**0.79**

a The model with higher F1 score has the better performance.

Using the selected RF model, we predicted the category for each project in the test dataset. The total donor support for CGH projects predicted by the RF model was $2.24 billion across 3 years, which was very close to the finding of $2.25 billion derived from coding and classification by humans. Table 2 presents the comparison between the predicted and human classified CGH funding amount, and the number of projects in each category in the test set.

**Table 2:** Comparing human and machine classified CGH funding (in million USD).

**CGH categories**	**Human classified funding (number of projects)^a^**	**Machine classified funding (number of projects)^b^**	**Absolute change between human classified and machine predicted funding^c^**	**Percentage change between human classified and machine predicted funding (%)^d^**
R&D of new health tools	293.64 (330)	316.07 (394)	22.43	7.64%
Development/harmonization of int. health regulations	22.02 (53)	19.17 (52)	2.85	−12.94%
Knowledge generation and sharing	222.27 (585)	220.50 (588)	1.77	−0.80%
Market shaping activities	611.52 (224)	628.95 (218)	17.43	2.85%
Epidemic and pandemic preparedness and response	276.48 (328)	271.72 (301)	4.76	−1.72%
Responses to marketing of unhealthful products	18.40 (60)	21.81 (54)	3.41	18.53%
Control of cross border disease movement	527.31 (310)	485.64 (310)	41.67	−7.90%
Health advocacy and priority setting	242.07 (203)	239.70 (202)	66.93	−0.98%
Promotion of aid effectiveness and accountability	32.14 (57)	35.69 (26)	3.55	11.05%
Total CGH	2,245.85 (2,150)	2,239.25 (2,145)	6.60	−0.29%
Not a Global function	905.36 (484)	911.98 (489)	6.62	0.73%

a The values in the bracket are Schäferhoff et al. manually classified projects in each CGH category in the test set. Funding in each category was calculated using manually classified projects in the test set.

b The RF model was used to predict the CGH category for each project in the test set. The values in the bracket are the machine predicted projects in each category. Funding in each CGH category was calculated using the machine predicted projects in the test set.

c Difference between machine predicted funding and human classified funding for each CGH category.

d Percentage change: (Machine classified funding - Human classified funding)/Human classified funding.

**Figure 2:**
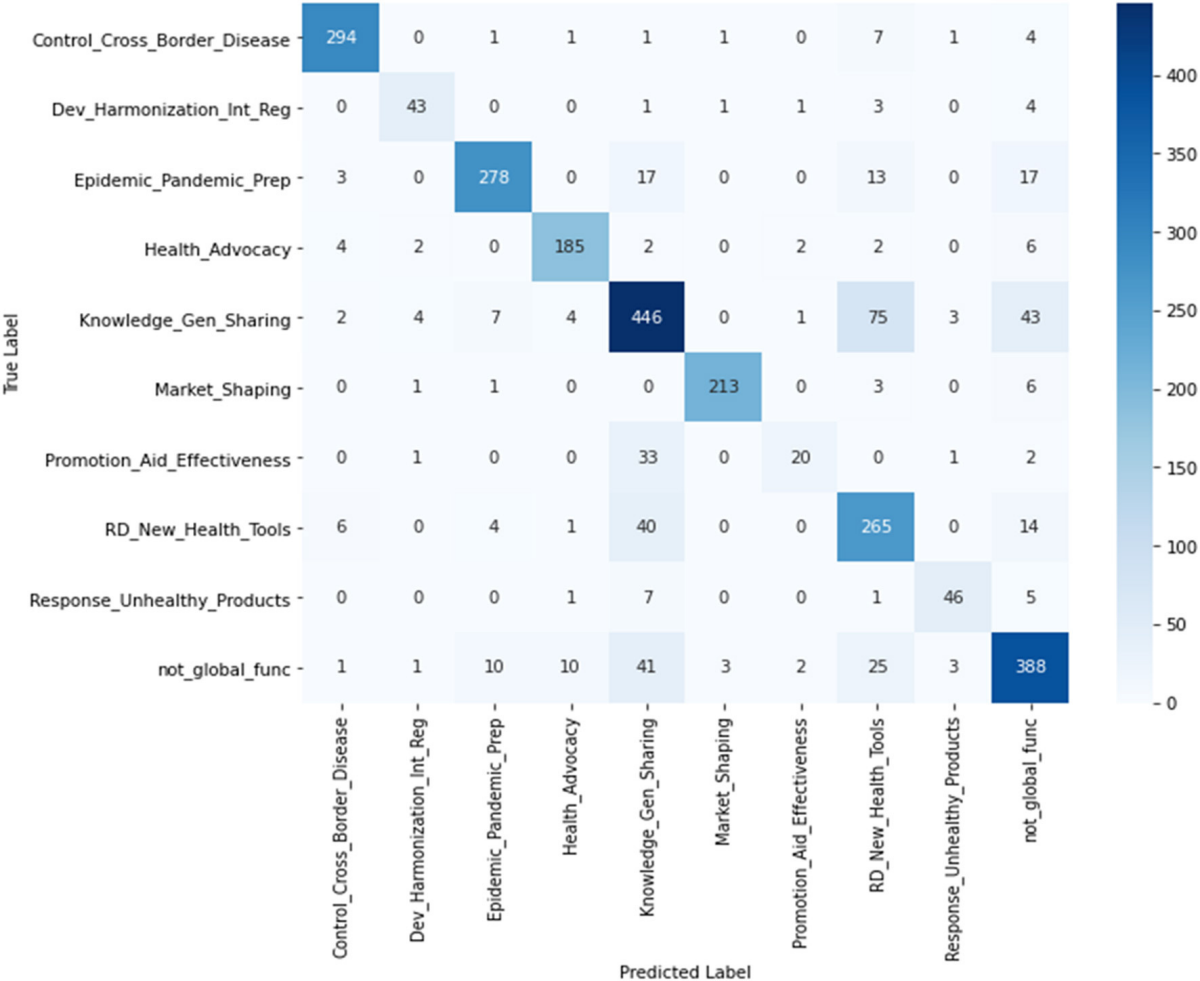
Confusion matrix to compare the human classified (True Label) and ML prediction (Predicted Label) projects.

In classification of the test data, the number of projects predicted by the ML model and the human coder were similar in most CGH categories. However, there were some misclassifications of projects from one CGH category into another. To capture this misclassification, a confusion matrix (Figure 2) was used, which provides two important pieces of information: (1) the number of projects from each category that were incorrectly classified, and (2) the categories into which these misclassified projects were placed. In Figure 2, “True Label” rows indicate the projects classified by a human classifier, and the “Predicted Label” columns list the projects classified by the ML model, disaggregated by CGH categories. The diagonal of the matrix shows the actual number of projects that matched the predicted projects in a category. For example, as per the human classifier, “promotion of aid effectiveness and accountability” had 57 projects. Of these 57 projects, only 20 projects (diagonal) were correctly predicted by the RF model, 33 projects were misclassified into “knowledge generation and sharing,” one project each was misclassified into “development/ harmonization of international health regulations” and “responses to marketing of unhealthful products,” respectively, and two projects were misclassified as “not a global function.” Figure 2 shows similar misclassifications for other CGH categories.

### Part 2: Application

The RF model was chosen for application to the 2019 data as it showed the best performance on the test data. Table 3 shows the number of predicted projects in the 2019 data classified into various CGH categories. The largest number of projects predicted by the model was in “Health advocacy and priority setting,” followed by “Control of cross border disease movement” and then “R&D of new health tools.” The funding for “Health advocacy and priority setting” at about $2 billion was many times more than the second highest funded category of “Control of cross border disease movement.” The funding for “Control of cross border disease movement” was about two-and -half times more than the funding for “R&D of new health tools.” The model predicted zero projects for “Promotion of aid effectiveness and accountability.” The total predicted funding for global CGH in 2019 was about $2.7 billion for a total of 730 CGH-related projects, less than the funding for CGH in 2017, 2015, and 2013 reported by Schäferhoff et al. (these authors estimated funding to be $4.4, $4.6, and $3.3 billion, respectively). As noted earlier, NCD projects were removed from the 2019 data as these projects were not available in the training dataset. Inclusion of projects in this category in the analysis could increase the CGH funding for 2019.

**Table 3:** Predicted projects and funding in different categories in 2019 by RF model.

**Categories**	**Predicted number of projects (*N*)**	**Predicted funding (in million USD)**
R&D of new health tools	116	112.17
Development/ harmonization of int. health regulations	6	5.35
Knowledge generation and sharing	102	34.01
Market shaping activities	27	45.12
Epidemic and pandemic preparedness and response	115	97.08
Responses to marketing of unhealthful products	8	13.56
Control of cross border disease movement	139	293.67
Health advocacy and priority setting	217	2,091.06
Promotion of aid effectiveness and accountability	0	0
Total CGH	730	2,692.02
Not a Global function	1,283	1,062.22
Total	**2,013**	**3,754.24**

Certain categories had lots of projects and yet the total funding for the category was relatively low. For example, the number of projects for “Knowledge generation and sharing” was 102, but the total funding was only $34.01 million. In comparison, the predicted number of projects for “R&D of new health tools” and “Epidemic and pandemic preparedness and response” were only a little higher at 116 and 115, respectively, but the total funding for these categories was $112.17, and 97.08 million, respectively.

## Discussion

This study explored the feasibility and accuracy of applying machine learning methods to classify funding for different types of global CGH. We applied four widely used machine learning algorithms—RF, XGBoost, SVM, and MNB—and found almost all the models performed well on the test data, with RF and XGBoost outperforming other models by small margins. We applied the RF model on test data and found the ML model predicted total donor support for CGH projects was very close to the human classified finding ($2.24 billion across 3 years for the ML model vs. $2.25 billion over 3 years for the human classification). However, the confusion matrix indicated that with the ML model there was misclassification in all CGH categories and categories with small numbers of projects tended to have more misclassifications. We further applied the trained and tested model to 2019 data and found that funding for CGH decreased in 2019, compared with 2017, 2015, and 2013, and health advocacy and priority setting, and control of cross border disease movement were the top two global CGH categories by the predicted number of projects.

The COVID-19 pandemic has changed the financing landscape for global health significantly. However, it also illustrated the importance of investing in CGH including R&D for pandemic control tools (e.g., vaccines, diagnostics, and antiviral drugs), pandemic preparedness, and control of cross-border diseases. We explored the application of ML in regularly tracking financing for CGH, which can be an essential step to inform the financing for CGH. We identified the decreasing trend of CGH between 2013 and 2019 and low financing for certain CGH sub-categories (such as R&D of new health tools, responses to marketing of unhealthful products and cross border disease control) called for more attention and efforts in fund raising.

Although 2019 data have not been manually coded and no information about CGH has been published, we have proven the prediction accuracy of our ML model on the test dataset. Therefore, our findings could suggest a funding trend for global CGH. Our study found that using the ML model to track financing for CGH was quick and required minimal labor to classify the projects. Additionally, we identified several opportunities to further improve the ML approach and automate the process of tracking CGH financing with better prediction.

At data collection and cleaning level, our analysis excluded certain projects from the training set, which limited the prediction power of ML models on those types of projects. First, we only focused on projects with English descriptions and dropping non-English projects may have resulted in the loss of important information on CGH funding. However, the goal of the current analysis was to show the feasibility of using ML in predicting CGH categories and financing, which we were able to successfully demonstrate. Therefore, in future research, models can be developed, using a similar ML pipeline described in the current research, to classify non-English projects and expand the scope of the analysis.

Second, we dropped two CGH categories with small numbers of projects to balance the number of projects for each CGH category— “Sharing of intellectual property” and “Responses to antimicrobial resistance (including to counterfeit drugs)”. However, these CGH categories, though in small numbers, are present every year in the OECD CRS database. Therefore, when we apply the model to new 2019 data, it would be advisable for us to incorporate the differences in the funding due to exclusion of these two categories as our model will only predict categories that it encountered in the training dataset. Having said this, given the small number of projects and funding in “Sharing of intellectual property” and “Responses to antimicrobial resistance” in the training data, exclusion of these two categories will not have a major impact on overall CGH funding levels in 2019.

Third, we had to remove NCD related projects from the 2019 data as our model was not trained on these projects. Since 2020, the OECD CRS started to include COVID-19 related projects. These projects can be added to the training dataset, and the ML model can then be retrained with the new categories in the database. Therefore, once we have a trained model, it can be quickly updated and retrained every few years to incorporate the changes/new project categories in the database. This could further improve the accuracy in prediction of CGH funding.

Moreover, in the current analysis, we included projects from the CRS database. The G-FINDER survey is another resource where additional projects on global CGH can be found (a subset of G-FINDER projects are captured in the CRS database, so we would have captured this subset). G-FINDER tracks the annual spending on product development for 33 poverty-related and neglected diseases (PRNDs) (32). Including G-FINDER data could increase the number of projects in some of the categories which had small number of projects in our training data.

As seen in Table 2, there were some differences in the financing for categories between ML prediction and human classification, which could be attributed to the misclassified projects. A small sample size is another reason for the low F1-score, which inhibits the model's classification ability, and often leads to misclassification of projects. To increase the sample size for the categories with small number of projects in the training dataset, we created synthetic projects but the lack of diversity of information from the small number of actual projects for the model to learn from affected the model's prediction power in certain categories. Using synthetic projects instead of actual projects to train the model could be a potential limitation of the study. However, collecting and including actual projects in categories with small numbers of projects could improve the model's learning and prediction power.

We also identified confounding project descriptions as another reason for the low F1-score, and the misclassification of projects. During our review on the project descriptions, we found that some project descriptions contained ambiguous or unclear information to classify projects into a certain category. For example, many projects in the “knowledge generation and sharing” category had text description that was very broad and included information that a machine and even a human classifier can easily group into multiple CGH categories. One way to address the issue of combined project descriptions is that instead of applying multiclass classification ML architecture, as used in the current research, one can implement a multilabel classification ML approach where a single project could be classified into multiple CGH categories.

## Conclusion

We developed and outlined a machine learning framework to automate the process of classifying health projects into different global CGH categories. Using this framework, we demonstrated that applying a machine learning approach in classification and tracking financing for global CGH is feasible, efficient, and can be done routinely. We also identified opportunities, based on the limitations of the study, for further improvement in the accuracy of the model. We found that the prediction accuracy can be increased by collecting more data for categories with small numbers of projects. Further studies can explore multilabel classification as some project descriptions are combinations of more than one global CGH category, and this might provide a more nuanced picture of CGH funding.

## Reflexivity statement

The authors include two females and three male and span multiple levels of seniority. While all of the authors specialize in global health and health policy research, one author has the expertise in machine learning. All authors have extensive experience conducting health policy research, including both qualitative, quantitative work in global health.

## Data availability statement

The original contributions presented in the study are included in the article/Supplementary material, further inquiries can be directed to the corresponding author/s.

## Ethics statement

This study used publicly available data without any individual information. Ethnical clearance is not applicable.

## Author contributions

SD, WM, and OO conceptualized this study. SD, KKM, and WM conducted analysis on the datasets. SD applied machine learning approaches with inputs from WM. SD and WM drafted the manuscript with inputs from all. All authors contributed to the article and approved the submitted version.

## Funding

This paper was part of the project Driving health progress during disease, demographic, domestic finance and donor transitions (the 4Ds): policy analysis and engagement with six transitioning countries funded by Bill and Melinda Gates Foundation (OPP1199624).

## Conflict of interest

Author MS was employed by Open Consultants. The remaining authors declare that the research was conducted in the absence of any commercial or financial relationships that could be construed as a potential conflict of interest.

## Publisher's note

All claims expressed in this article are solely those of the authors and do not necessarily represent those of their affiliated organizations, or those of the publisher, the editors and the reviewers. Any product that may be evaluated in this article, or claim that may be made by its manufacturer, is not guaranteed or endorsed by the publisher.
