# Supplementary material for: Tracking financing for global common goods for health: A machine learning approach using natural language processing techniques
Source: Front Public Health. 2022 Nov 17;10:1031147. doi: 10.3389/fpubh.2022.1031147 (PMC9712779; doi:10.3389/fpubh.2022.1031147)
Supplement: Supplementary file 1 [file Table_1.DOCX]

**Annexures**

**Annex 1****:** **CRS purpose codes assessed to create the 2019 dataset**

| CRS purpose code | | Description |
| --- | --- | --- |
| 121 (Health, General) | 12110 | Health policy and administrative management |
|  | 12196 | Health statistics and data |
|  | 12181 | Medical education/training |
|  | 12182 | Medical research |
|  | 12191 | Medical services |
| 122 (Basic Health) | 12220 | Basic health care |
|  | 12230 | Basic health infrastructure |
|  | 12240 | Basic nutrition |
|  | 12250 | Infectious disease control |
|  | 12261 | Health education |
|  | 12262 | Malaria control |
|  | 12263 | Tuberculosis control |
|  | 12281 | Health personnel development |
| 160 (Other Social Infrastructure & Services) | 16064 | Social mitigation of HIV/AIDS |
| 720 (Emergency Response) | 72010 | Material relief assistance and services |
|  | 72040 | Emergency food assistance |
|  | 72050 | Relief co-ordination and support services |
| 730 (Reconstruction Relief & Rehabilitation) | 73010 | Immediate post-emergency reconstruction and rehabilitation |
| 740 (Disaster Prevention & Preparedness) | 74020 | Multi-hazard response preparedness |

Source: OECD CRS database ^1^

Note: These were the same set of codes used by Schäferhoff et al. to create their dataset

**Annex 2 Global CGH definitions**

| **Global CGH sub-functions** | **Activities included** |
| --- | --- |
| R&D of new health tools | - Product development for poverty-related and neglected   diseases (as defined by G-FINDER) |
| Development and harmonization  of international health  regulations | - Global and regional technical guidelines, norms, standards, and framework conventions - Development and harmonization of global and regional norms - Guidelines and pre-qualification related to R&D |
| Knowledge generation and  sharing | - Global and regional research, e.g., systematic reviews - Global and regional statistics/data (e.g., burden of disease studies, the WHO Global Health Expenditure Database) - National studies that contribute to the global evidence base (e.g., impact evaluations, policy & implementation research) - Distribution of knowledge through global, regional, and South-to-South networks, partnerships, programs, conferences, and other mechanisms |
| Sharing of intellectual property | - Activities that help make products and knowledge available to the poor, such as patent pooling and free licensing |
| Market-shaping activities | - Global, regional, and national market-shaping activities to drive down costs of health technologies, including bulk procurement - Capacity building for local manufacturers |
| Epidemic and pandemic preparedness and response | - R&D for medical countermeasures (MCMs) for infections with pandemic potential (WHO’s R&D Blueprint diseases plus pandemic influenza) - Global, regional, and national outbreak surveillance systems - Global, regional, and national surge capacity (for manufacture of vaccines and other MCMs) Global, regional, and national stockpiles of MCMs - Policies/regulations to manage outbreaks - Pandemic insurance mechanisms |
| Responses to antimicrobial  resistance (AMR) | - Activities targeting AMR - Activities addressing TB drug resistance, including treatment of multidrug-resistant tuberculosis (MDR TB) and extensively drug-resistant tuberculosis (XDR TB) |
| Responses to marketing of  unhealthful products | - Global, regional, national activities in response unhealthful products (e.g., alcohol and tobacco) |
| Control of cross-border disease  movement | - Global, regional, and national eradication and elimination activities, including for polio and malaria - Cross-border and regional disease programs - Biosecurity/biosafety projects |
| Health advocacy and priority  setting | - Development of global and regional plans and strategies - Leadership/stewardship for specific diseases, conditions, or health overall - Convening for consensus building for strategy and policy - Cross-sectoral advocacy and advocacy for the health of neglected, marginalized, and conflict-affected groups |
| Promotion of aid effectiveness  and accountability | - Global and regional accountability for results and financing - Global and regional activities geared towards donor harmonization |

Source: Schäferhoff et. al.^2^

**Annex 3: Number of projects in each CGH category before data/text cleaning step**

Source: Authors calculation using data obtained from Schäferhoff et. al.^2^

**Annex 4: Number of projects in each CGH category after data/text cleaning step**

Source: Authors calculation using data obtained from Schäferhoff et. al.^2^

# **REFERENCES**

1. DAC and CRS code lists - OECD [Internet]. [cited 2022 May 9]. Available from: https://www.oecd.org/dac/financing-sustainable-development/development-finance-standards/dacandcrscodelists.htm

2. Schäferhoff M, Chodavadia P, Martinez S, McDade KK, Fewer S, Silva S, et al. International Funding for Global Common Goods for Health: An Analysis Using the Creditor Reporting System and G-FINDER Databases. Health Syst Reform. 2019 Oct 2;5(4):350–65.
